# Monoamine Oxidase A Contributes to Serotonin—But Not Norepinephrine-Dependent Damage of Rat Ventricular Myocytes

**DOI:** 10.3390/biom13061013

**Published:** 2023-06-19

**Authors:** Jonas Knittel, Nadja Itani, Rolf Schreckenberg, Jacqueline Heger, Susanne Rohrbach, Rainer Schulz, Klaus-Dieter Schlüter

**Affiliations:** Physiologisches Institut, Justus-Liebig-Universität, 35392 Gießen, Germany

**Keywords:** oxidative stress, mitochondria, ageing

## Abstract

Serotonin effects on cardiac hypertrophy, senescence, and failure are dependent either on activation of specific receptors or serotonin uptake and serotonin degradation by monoamine oxidases (MAOs). Receptor-dependent effects are specific for serotonin, but MAO-dependent effects are nonspecific as MAOs also metabolize other substrates such as catecholamines. Our study evaluates the role of MAO-A in serotonin- and norepinephrine-dependent cell damage. Experiments were performed in vivo to study the regulation of *MAOA* and *MAOB* expression and in vitro on isolated cultured adult rat ventricular cardiomyocytes (cultured for 24 h) to study the function of MAO-A. *MAOA* but not *MAOB* expression increased in maladaptive hypertrophic stages. Serotonin and norepinephrine induced morphologic cell damage (loss of rod-shaped cell structure). However, MAO-A inhibition suppressed serotonin-dependent but not norepinephrine-dependent damages. Serotonin but not norepinephrine caused a reduction in cell shortening in nondamaged cells. Serotonin induced mitochondria-dependent oxidative stress. In vivo, *MAOA* was induced during aging and hypertension but the expression of the corresponding serotonin uptake receptor (*SLC6A4*) was reduced and enzymes that reduce either oxidative stress (*CAT*) or accumulation of 5-hydroxyindolacetaldehyde (*ALDH2*) were induced. In summary, the data show that MAO-A potentially affects cardiomyocytes’ function but that serotonin is not necessarily the native substrate.

## 1. Introduction

Serotonin (=5-Hydroxytryptamin, 5-HT) is a phylogenetically old neurotransmitter that is nearly ubiquitously expressed in all species [1]. In vertebrates, 5-HT affects blood pressure, thrombosis, and central neurological functions such as sleep, appetite, nociception, and spirit [2,3,4,5,6,7]. Effects of 5-HT on cardiomyocytes have long been described [8]. They can be either rather specific or unspecific. Specific functions of 5-HT on cardiomyocytes depend on activation of specific receptors., The receptors 5-hydroxytryptamine receptor 2B, encoded by the *HTR2B* gene, and 5-hydroxytryptamine receptor 2A, encoded by the gene *HTR2A*, are best investigated in the cardiac field [9,10]. Nonspecific effects of 5-HT are linked to 5-HT uptake via the serotonin transporter 5-HTT, encoded by the gene *SLC6A4*, and serotonin metabolism by monoamine oxidases (MAO)s that metabolize 5-HT to 5-hydroxyindolacetaldehyde. Two different isoforms of MAO are known, namely, MAO-A and MAO-B. Both are located at the outer mitochondrial membrane. The isoforms differ in substrate specificity. Among them, 5-HT and norepinephrine are considered as specific for MAO-A and β-phenylethlyamine (PEA) for MAO-B. MAOs require flavin–adenin–dinucleotid (FAD) as a cofactor that is reduced by the reaction and subsequently reoxidized by O_2_ and H_2_O, generating hydrogen peroxide [11,12]. Hydrogen peroxide is subsequently detoxified by catalase, encoded by the gene *CAT* whereas 5-hydroxyindolacetaldehyde is further metabolized to 5-hydroxyindolacetate. This reaction is catalyzed by aldehyde–dehydrogenase, encoded by the gene *ALDH2*. MAO-A also metabolizes catecholamines, such as norepinephrine. In case of norepinephrine, the substrate is taken up by an organic cation transporter (OCT). It seems that 5-HT exerts specific (receptor-dependent) effects at lower concentration of 5-HT and MAO-A-dependent intracellular effects at higher concentration of 5-HT [13].

Cardiomyocytes contain 5-HT, 5-HT receptors, and MAOs [14,15]. Cardiomyocytes from evolutionary near species such as rats and mice express different isoforms. The reason for expression of different isoforms is not clear. In rat hearts, MAO-A is preferentially expressed [16]. The function of MAO-A in rat cardiomyocytes remains to be established. On the one hand, MAO-A affects substrate metabolism and improves the use of glucose [17]. In this regard it is important that MAO-A is induced under conditions of cardiac hypertrophy that goes along with a metabolic switch to preferential use of carbohydrates rather than fatty acids. MAO-A shares these associations with other mitochondrial proteins, such as uncoupling protein 2 (UCP2), a protonophore located at the inner mitochondrial membrane. In the case of UCP2, which potentially reduces oxidative stress, a downregulation is associated with metabolic shift to glucose consumption [18]. In both cases, upregulation of MAO-A and downregulation of UCP2, oxidative stress may occur. This may trigger the metabolic shift. A possible common target may be HIF1α, which can be activated in an ROS-dependent way and affect the expression of glucose transporters [19,20]. On the other hand, there are several reports that high MAO-A activity causes cellular damage related to oxidative stress [21,22,23,24]. However, 5-HT can protect cardiomyocytes via receptor-dependent signaling pathways [9].

Similar to 5-HT, norepinephrine, another MAO-A substrate, can be taken up by cardiomyocytes via OCT [24]. It has recently been suggested that upregulation of MAO-A during heart failure will accelerate intracellular catecholamine degradation, thereby inhibiting a direct stimulation of β-adrenergic receptors at the sarcoplasmatic reticulum (SR). Such an interaction between β-adrenoceptors and sarcoplasmic reticulum was closely linked to phospholamban phosphorylation and calcium filling of the SR [24]. It is currently unclear whether this affects cell structure and function in cardiomyocytes exposed to excessive catecholamines. Nevertheless, pharmacological inhibition of MAO-A should increase the intracellular concentration of norepinephrine; thereby, the calcium content of the SR should increase improving contractility. Furthermore, 5-HT and catecholamine-dependent effects are further linked on the level of receptors as receptors for both ligands can undergo heterologous receptor dimerization, linking the activation of β_2_-adrenoceptors from G_αs_- to G_αi_-dependent pathways [25].

In this study, we tried to address some of the open questions that still occur concerning the role of MAOs in cardiomyocytes. At first, we addressed the question of whether MAO upregulation in the context of acute (experimental) pressure overload is specific for *MAOA*, valid for the left and right ventricle, and whether potential effects of 5-HT can be demonstrated in cardiomyocytes from right and left ventricles. Second, we addressed the questions of whether MAO-A-specific substrates such as 5-HT favor oxidative stress and whether this triggers the cell-damaging effects of high concentrations of 5-HT. Third, we addressed the question of whether chronic pressure overload and ageing commonly affect the expression of MAOs and proteins linked to uptake and metabolism of MAO substrates. Finally, we compared findings with 5-HT to those with norepinephrine, as norepinephrine metabolism by MAOs has most recently been linked to calcium handling in cardiomyocytes and function. Supplementary Figure S1 gives an overview of the questions addressed here.

## 2. Materials and Methods

### 2.1. Ethical Concerns

All animals were maintained under conditions that conform to the Guide for the Care and Use of Laboratory Animals (NIH Publication No. 85-23, revised 1996). The study was endorsed by the institutional animal care committee of the Justus-Liebig-University Giessen, and the approval of animal investigation was contracted by the local authorities (RP Giessen, V54-19c2015h GI20/10 Nr. G 12/2018). For details for housing and testing of the MAO-B mice, see [26]. Adult Wistar rats (males, 200–350 g) were ordered from Janvier Labs (Le Genest Saintz Isles, France). Protocols for organ removal were approved by the Justus-Liebig-University (permission number 666_M and 561_M). Analysis of MAO-A and MAO-B expression in rat hearts with aortic banding and pulmonary artery banding (*n* = 30 rats) was performed on tissue material derived from a previous study [27]. These experiments were registered under number G14-2017.

### 2.2. Cell Isolation

Cardiomyocytes (adult rat ventricular myocytes, ARVM) were isolated from rats (*n* = 4 rats) or mice hearts (*n* = 3 mice) as described in great detail in [28,29]. Briefly, hearts were extracted from anaesthetized (isoflurane) rodents after cervical dislocation and immediately transferred to a Langendorff system. The tissue was prepared for isolation by calcium-free perfusion with collagenase, and subsequently minced and transferred to calcium-containing buffer again. The remaining calcium-tolerant myocytes were attached to culture dishes (Falcon, 3004) and maintained in serum-free CCT medium (M199 with supplementation of creatinine, carnitine, and taurine).

### 2.3. Samples from Ageing and Hypertensive Rats

Samples from left ventricular tissue were used to quantify the expression of genes related to 5-HT metabolism or signaling. These samples were obtained from rats previously used for other studies as well [30,31,32]. For details of animal characteristics and blood pressure, please see [27,31]. In total, 18 female normotensive rats were analyzed for the ageing part and 12 female rats (normotensive or hypertensive) were analyzed in the comparison between normotension and hypertension.

### 2.4. Mitochondria Isolation

For mitochondria isolation, the whole left ventricle was minced in isolation buffer containing sucrose (250 mM), HEPES (10 mM), and EGTA (1 mM) and homogenized with a 15 mL glass Potter as described before [26]. In total, four rats were used for mitochondria isolation.

### 2.5. Detection of ROS

Hydrogen peroxide was detected by Amplex Ultra Red (A36006; Invitrogen, Waltham, MA, USA) reagent. A total of 25 µg of freshly isolated mitochondria were used. Fluorescence was measured continuously for ten minutes with excitation and emission wavelengths of 565 and 581 nm, respectively, in a Care Eclipse spectrophotometer (Agilent, Santa Clara, CA, USA). Glutamate (5 mM) and malate (2.5 mM) were used as substrates for complex I, as described before [26].

### 2.6. Cardiomyocytes Contraction

Cell shortening was analyzed as described before [33]. Basal contraction parameters were analyzed at 2 Hz for one min at room temperature. Analysis of contraction was performed using a cell-edge-detection system. Data were registered every 15 s and the mean of these four measurements was used. Shortening of cells was calculated as shortening amplitude (in µm) normalized to the diastolic cell length (in µm) and expressed as %. Experiments are based on isolation of myocytes from 11 mice and 30 rats.

### 2.7. Cell Structure

Cultures were evaluated after 24 h by light microscopy. The number of rod-shaped cells per area was counted. Round cells or cells with unusual appearance (for definition see [34]) were not counted. Experiments are based on nine preparations.

### 2.8. Western Blot

Tissues or cells were lysed in lysis buffer as described before in detail. Volumes of equal (40 µg) proteins were separated by electrophoresis on 10% SDS gels and transferred to nitrocellulose membranes. Western blots were performed using a standard protocol, with specific primary antibodies against MAO-A (Ab126751, Abcam, Cambridge, UK), MAO-B (#1821, SIGMA, St. Louis, MO, USA), and GAPDH (#5G4Mab6C5, Hy Test Ltd., Turku, Finland) and HRP-conjugated goat-anti-rabbit IgG. Immunoreactive bands were detected using SuperSignal West Femto Maximum Sensitivity Substrate (Pierce, Rockford, IL, USA). Protein bands were quantified by Quantity One software (Version 4.6.9; Bio-Rad Laboratories, Hercules, CA, USA). GAPDH was used as loading control. Experiments are based on analysis of four rats.

### 2.9. PCR Analysis

Total RNA from ventricular tissues was isolated using peqGOLD TriFast according to the manufacturer’s protocol as described before [31]. DNAse was used to remove DNA contamination. One µg of RNA was used to synthesize cDNA using Superscript RNase H reverse transcriptase and oligo(dt) as primers. The sequences of the primers used in this study are indicated in Supplement Table S1. Quantification (2^−ΔΔCT^ method) was analyzed as described before [35]. Thresholds of the gene of interest were normalized to the mean thresholds of three housekeeping genes (*B2M*, *HRPT1*, *RPL32*).

### 2.10. Statistics

Data are expressed as box-and-whiskers plots representing the full range of all samples (whiskers) and the 25%, 50%, and 75% quartiles as boxes. Data were analyzed by two-sided one-way ANOVA with Student–Newman–Keuls post hoc analysis or Kruskal–Wallis test with Bonferroni post hoc analysis (correction for multiple testing). In cases where two samples were compared, *t*-tests or Mann–Whitney U-tests were used. The use of the different tests is indicated in the figure legends. Exact *p*-values are given. Direct comparison between two groups is stated as effect size with 95% confidence interval based on Cohen’s analysis.

## 3. Results

### 3.1. Effect of Pressure Overload on the Expression of MAOA and MAOB in Rats Hearts

**Figure 1:**
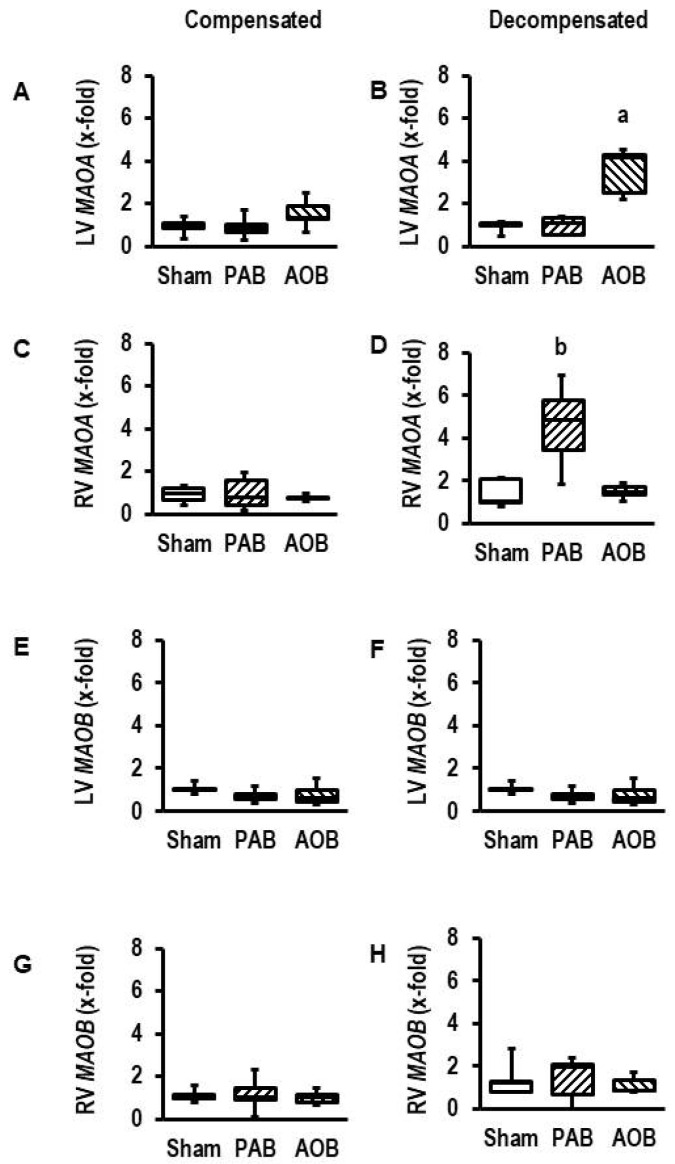
Expression of MAO isoforms in rat hearts exposed to pressure overload by aortic banding (AOB) and pulmonary artery banding (PAB). (**A**) Expression of *MAOA* in the left ventricle in hearts from AOB and PAB rats with still normal cardiac function (compensated state). (**B**) Expression of *MAOA* in the left ventricle in hearts from AOB and PAB rats with reduced cardiac function (decompensated state). (**C**) Expression of *MAOA* in the right ventricle in hearts from AOB and PAB rats with still normal cardiac function (compensated state). (**D**) Expression of *MAOA* in the right ventricle in hearts from AOB and PAB rats with reduced cardiac function (decompensated state). (**E**) Expression of *MAOB* in the left ventricle in hearts from AOB and PAB rats with still normal cardiac function (compensated state). (**F**) Expression of *MAOB* in the left ventricle in hearts from AOB and PAB rats with reduced cardiac function (decompensated state). (**G**) Expression of *MAOB* in the right ventricle in hearts from AOB and PAB rats with still normal cardiac function (compensated state). (**H**) Expression of *MAOB* in the right ventricle in hearts from AOB and PAB rats with reduced cardiac function (decompensated state). Data are full ranges (whiskers) with median and 25 and 75% quartiles (boxes) (*n* = 5 hearts each). a, *p* = 0.000077 in two-sided ANOVA with AOB > Sham and PAB in Student–Newman–Keuls post hoc analysis; b, *p* = 0.002 in two-sided ANOVA with PAB > Sham and AOB in Student–Newman–Keuls post hoc analysis; *p* > 0.05 (two-sided ANOVA) for all (**A**–**H**).

At first, we addressed the question of whether *MAOA* or *MAOB* is induced by pressure overload in left and right ventricles of rats. The analysis was performed on tissue samples from rats used previously to study transcriptional adaptation at adaptive and maladaptive hypertrophy [28]. Here, we added the analysis of *MAOA* and *MAOB* expression in left and right ventricles. The data showed that *MAOA* but not *MAOB* is induced in the failing state in both ventricles but not in the compensatory state (Figure 1). *MAOA* was induced in the left ventricle of aortic banding rats in the decompensated state (effect size: 3.288; 95% confidence interval (CI: 1.234–5.261; *p* = 0.005) and in the right ventricle of pulmonary artery banding rats in the decompensated state (effect size: 2.138; 95% CI: 0.489–3.712; *p* = 0.010).

### 3.2. Cardiac Expression of MAO-A and MAO-B in Rats and Mice

**Figure 2:**
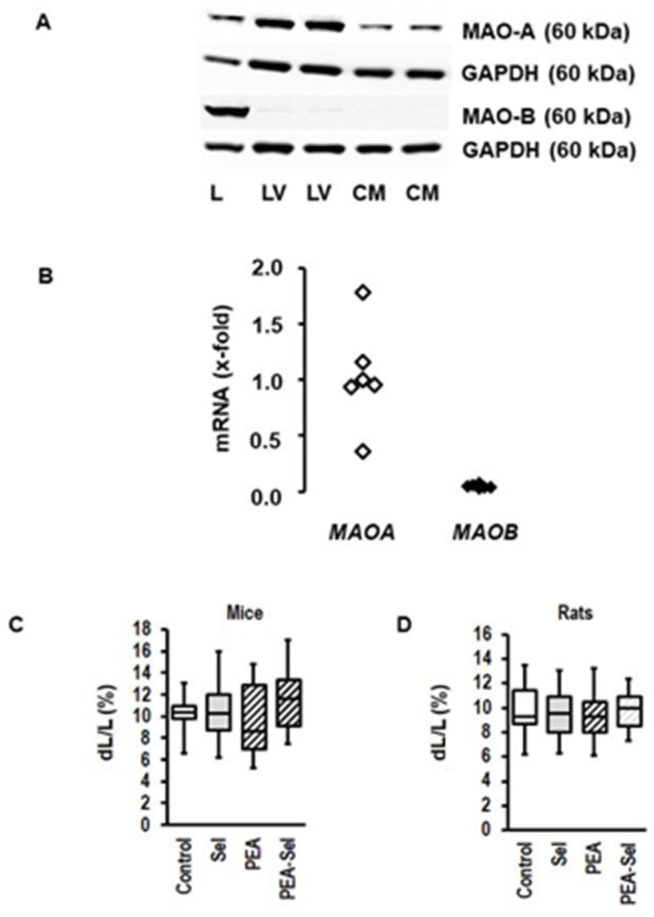
Expression of MAO isoforms in rat heart and myocytes and effect of MAO-B activation on mouse and rat myocytes. (**A**) Western blot indicating the expression of MAO-A and MAO-B in the liver (L), left ventricle (LV), and cardiomyocytes (CM) from rat hearts. GAPDH was used as loading control. (**B**) mRNA expression in rat hearts of the genes (*MAOA*; *MAOB*) encoding for MAO-A and MAO-B. The data are normalized to beta-2-microglobulin (b2m) as a housekeeping gene. The mean of *MAOA* expression is set as 1. (**C**) Load-free cell shortening is expressed as % shortening amplitude (dL) normalized to the diastolic cell length (L). Data are full ranges (whiskers) with median and 25 and 75% quartiles (boxes). Cells (isolated ventricular myocytes from mice) were incubated with selegiline (Sel; 1 µM; *n* = 26), β-phenylethylamine (PEA, 250 µM; *n* = 18), or combinations thereof (*n* = 27) for 24 h. Untreated controls (*n* = 18) were used to control the quality of the preparation. Cells were stimulated at 2 Hz and cell shortening was monitored by a line camera. (**D**) Similar experiment to C, but with rat myocytes (ARVM; *n* = 36 each).

In contrast to other tissues, such as the liver, the rat heart and ARVM express nearly exclusively MAO-A (Figure 2A). The levels of protein expression are accompanied by a similar profile of *MAOA* and *MAOB* expression in cardiac tissue (Figure 2B). The expression level of *MAOB* in nonmyocytes accounted for only 8 ± 3% (*p* < 0.001; *n* = 4) of that of *MAOA* in myocytes. When cardiomyocytes were exposed to PEA, a substrate preferentially utilized by MAO-B and inducing oxidative stress in mice myocytes [27], this did not affect load-free cell shortening in mice myocytes, a surrogate parameter of cardiomyocytes’ contractility (Figure 2C). Mouse myocytes were previously positive-tested for MAO-B expression [26]. There was a small tendency to reduced cell shortening levels in presence of PEA (effect size 0.277; 95% confidence interval (CI): −0.382–0.932) that was nullified by selegiline, an MAO-B inhibitor (Figure 2C); however, increasing the concentration of PEA did not exert a significant effect (controls: ΔL/L (%): 9.32 ± 3.82 (*n* = 17, *N* = 3); PEA (1 mM: 9.33 ± 3.10 (*n* = 38, *N* = 3; *p* = 0.990). As expected from the expression profile, PEA did not affect load-free cell shortening in ARVM (Figure 2D). In summary, molecular (protein and mRNA) and functional data exclude a relevant role for MAO-B in ARVM.

### 3.3. Effect of 5-HT on Load-Free Cell Shortening in Mouse Myocytes and ARVM

**Figure 3:**
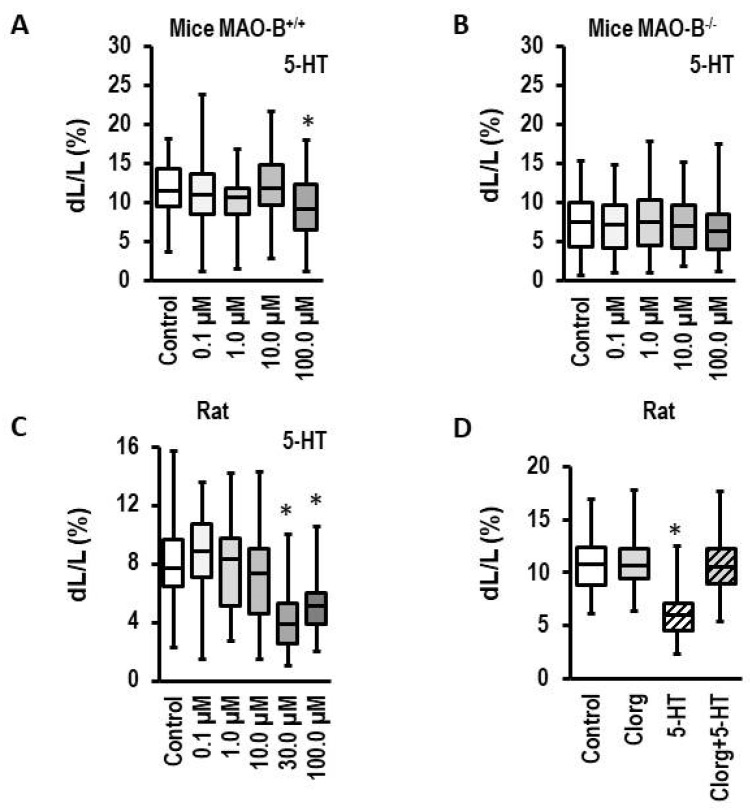
Effect of 5-HT on load-free cell shortening. (**A**–**C**) Concentration–response curves for myocytes from MAO-B expressing mice myocytes (MAO-B^+/+^, *n* = 54–72), MAO-B knockout myocytes (MAO-B^−/−^, *n* = 65–72), and ARVM (*n* = 28–45). Cells were exposed to 5-HT for 24 h and cell shortening was subsequently analyzed as in Figure 1. (**D**) Effect of clorgyline (Clorg, 1 µM), 5-HT (100 µM), and combinations thereof (*n* = 45–108). Data are full ranges (whiskers) with median and 25 and 75% quartiles (boxes). *; *p* < 0.05 vs. control; Kruskal–Wallis test with Bonferroni (correction for multiple testing) post hoc analysis.

In contrast to PEA, 5-HT is preferentially metabolized by MAO-A. As expected, 5-HT did only minimally affect load-free cell shortening of mice myocytes at the highest concentration tested here (100 µM; effect size 0.740 (CI: 0.389–1.088); Figure 3A). Importantly, this response was attenuated in myocytes isolated from MAO-B knockout mice (effect size 0.221 (CI: −0.107–0.549); Figure 3B). These data confirm that 5-HT is a minor substrate for MAO-B. However, isolated myocytes from knockout mice had a lower basal contractility (Figure 3A,B). In contrast, high MAO-A expressing ARVM showed a strong functional impairment at already 30 µM (effect size: 1.341 (CI: 0.765–1.885); Figure 3C). This effect was attenuated by clorgyline, an MAO-A-specific inhibitor (effect size: 0.108 (CI: −0.159–0.375); Figure 3D). In summary, molecular (protein and mRNA) and functional data support a relevant role for MAO-A in rat but not mouse heart.

### 3.4. Effect of 5-HT on Structural Integrity of ARVM

**Figure 4:**
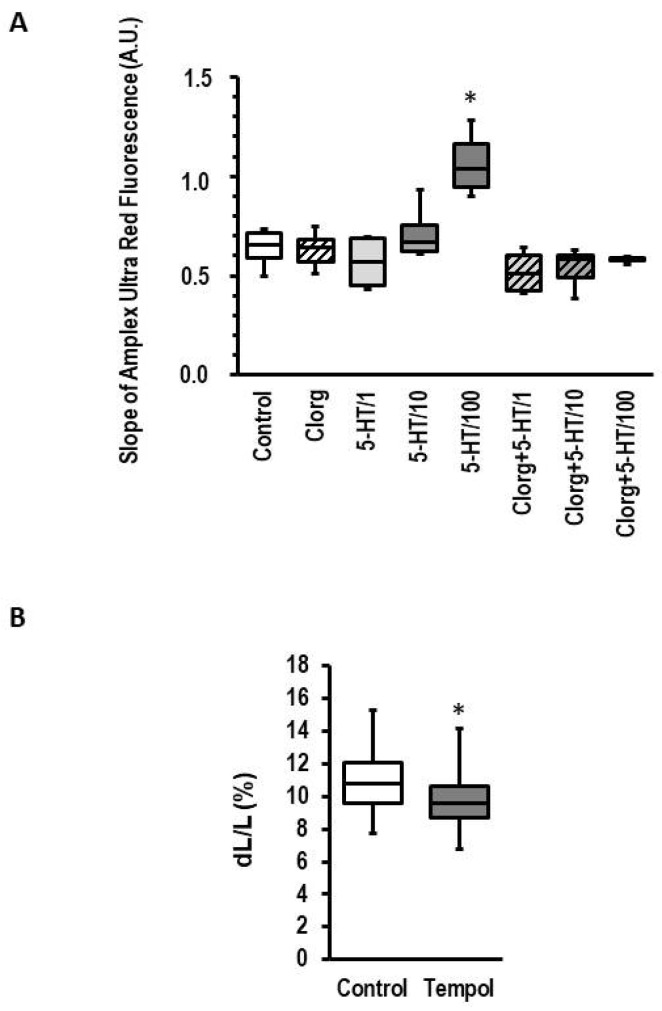
Effect of 5-HT on mitochondrial ROS production and ROS on cell shortening. (**A**) Rat mitochondrial ROS production. Mitochondria from rat hearts were isolated and ROS production was measured in the presence of complex I substrate, with 5-HT (concentration 1–100 µM) and/or clorgyline (1 µM) and 5-HT. Data are full ranges (whiskers) with median and 25 and 75% quartiles (boxes). *; *p* < 0.05 vs. control; Kruskal–Wallis test with Bonferroni (correction for multiple testing) post hoc analysis; *n* = 4–8. (**B**) Effect of Tempol (1 mmol/L) on cell shortening. Data are full ranges (whiskers) with median and 25 and 75% quartiles (boxes). *; *p* = 0.000192 vs. control; Student’s *t*-test; *n* = 54.

MAO-A-dependent effects have been linked to oxidative stress because the transformation of 5-HT to 5-hydroxyindolacetaldehyde generates hydrogen peroxide as a byproduct. MAOs are located at the outer mitochondrial membrane. Therefore, stimulation of either MAO-A or MAO-B should generate H_2_O_2_ that can be analyzed by Amplex Ultra Red. In isolated mitochondria from rat hearts, 5-HT concentration dependence increased the slope of Amplex Ultra Red fluorescence, indicating the production of reactive oxygen species (ROS) (Figure 4). More important, the effect of 5-HT on ROS production was attenuated by copresence of the MAO-A inhibitor clorgyline (Figure 4). To further investigate whether H_2_O_2_ is part of the MAO-dependent effect on cell shortening, we used tempol, a superoxide dismutase mimeric that generates H_2_O_2_ by detoxifying superoxide [36]. We found that exposure of cardiomyocytes to tempol for 24 h reduced cell shortening by 11.0% (Figure 4B; effect size 0.744 (CI: 0.352–1.132).

**Figure 5:**
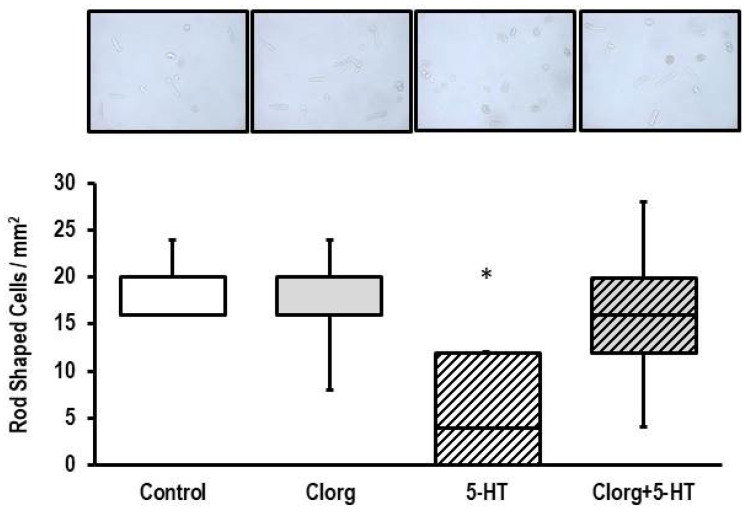
Effect of 5-HT on cell structure. (**Top**) Original photographs obtained from cultures exposed to clorgyline (1 µM), 5-HT (100 µM), or combinations thereof for 24 h (*n* = 5 each). For greater visibility of rod-shaped and round cells, see Supplementary Information. Data are expressed as the number of rod-shaped cells per mm^2^. Data are full ranges (whiskers) with median and 25 and 75% quartiles (boxes). *; *p* < 0.05 vs. control; Kruskal–Wallis test with Bonferroni (correction for multiple testing) post hoc analysis.

Overnight exposure of ARVM to 5-HT damaged the cells, as visualized by a strong decrease in the amount of rod-shaped cells (Figure 5). Again, this effect was attenuated by clorgyline (Figure 5).

### 3.5. Effect of 5-HT on ARVM Isolated from Either the Right or Left Ventricle

Differences in ROS scavenging contribute to stress adaptation between the left and right ventricle. Therefore, we analyzed the expression of MAO-A in both ventricles and the responsiveness of myocytes isolated from both ventricles to 5-HT. Although *MAOA* is constitutively expressed in left and right ventricles, the expression of *MAOA* in right ventricles was lower than that of the left ventricle (43 ± 12%; *p* = 0.041, *n* = 7–8; effect size: 1.196 (CI: 0.065–2.289).

**Figure 6:**
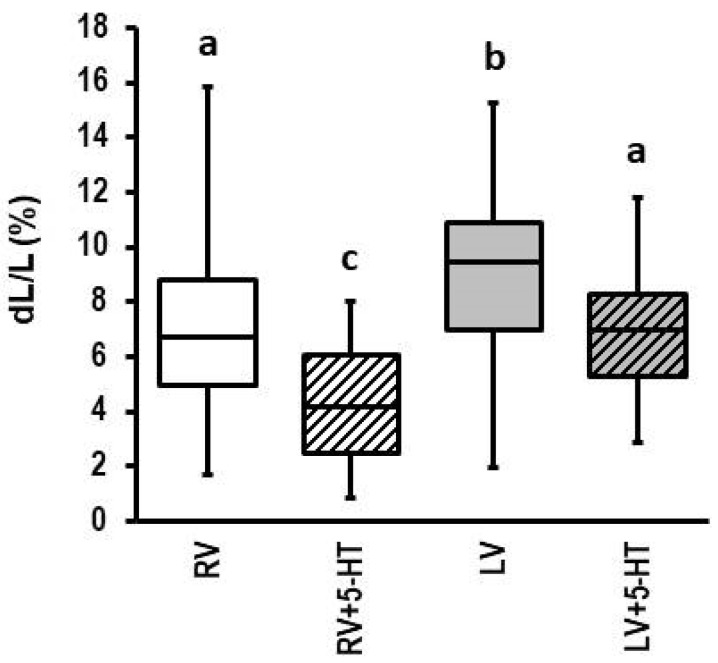
Effect of 5-HT on cell shortening of cardiomyocytes isolated from right (RV) or left (LV) ventricle. Cells (*n* = 27 (RV or 54 (LV) were exposed to 5-HT (100 µM) for 24 h and cell shortening was subsequently analyzed as in Figure 1. Data are full ranges (whiskers) with median and 25 and 75% quartiles (boxes). One-way ANOVA with Student–Newman–Keuls post hoc analysis. Different letters indicate group differences with *p* < 0.05.

Consistent with common findings, isolated myocytes from the right ventricle displayed a smaller load-free cell shortening than those of the left ventricle (Figure 6). However, 5-HT reduced load-free cell shortening in both ventricles, although the effect was stronger in myocytes isolated from the right ventricle (Figure 6; effect size left ventricle: 0.689; CI: 0.299–1.076; *p* = 0.00055); effect size right ventricle: 1.082 (CI: 0.505–1.650; *p* = 0.000217)).

### 3.6. Effect of Ageing and Hypertension on the Expression of MAOA and Genes Required for 5-HT Metabolism

**Figure 7:**
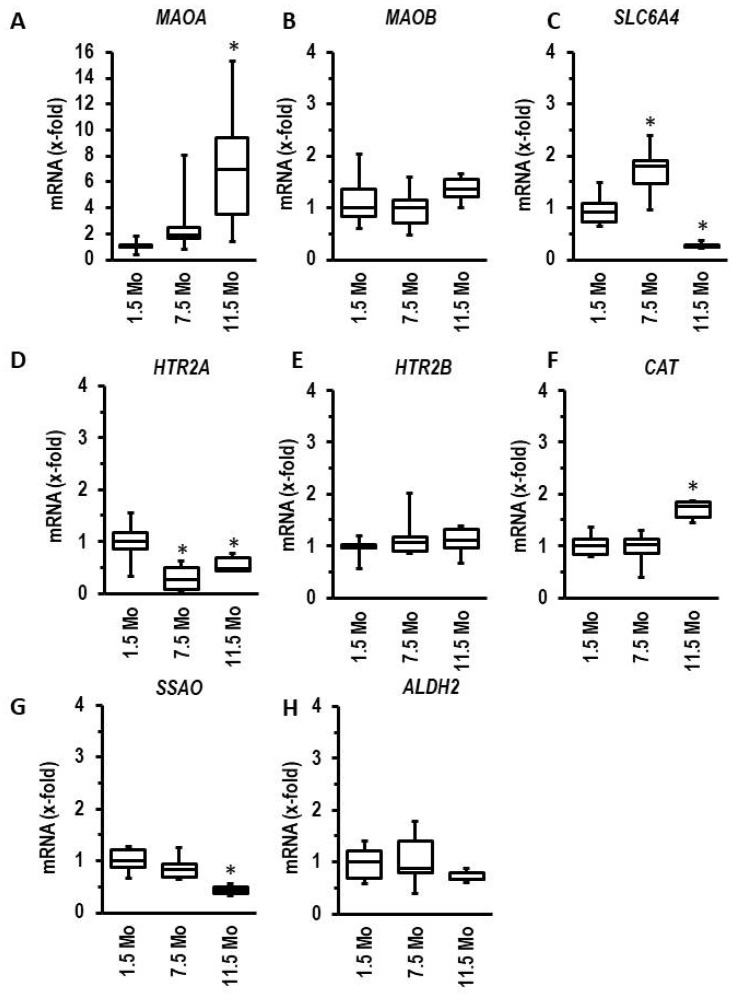
Age-dependent regulation of left ventricular mRNA expression of genes involved in 5-HT metabolism or signaling ((**A**): *MAOA*, (**B**): *MAOB*; (**C**): *SLC6A4*; (**D**): *HTR2A*; (**E**): *HTR2B*; (**F**): *CAT*; (**G**): *SSAO*; (**H**): *ALDH2*). Left ventricular tissue was dissected from rats at the age of 1.5, 7.5, or 11.5 months (*n* = 6 each). Data are full ranges (whiskers) with median and 25 and 75% quartiles (boxes). One-way ANOVA with Student–Newman–Keuls post hoc analysis or Kruskal–Wallis test with Bonferroni (correction for multiple testing) post hoc analysis where appropriate. * *p* < 0.05 vs. 1.5 months old.

The aforementioned experiments with myocytes from right and left ventricles indicate that a higher expression of *MAOA* alone is not sufficient to indicate a stronger effect of 5-HT on the myocardium. Therefore, in normotensive rats, we analyzed the effect of ageing on the expression of *MAOA* and genes linked to 5-HT metabolism and 5-HT receptors. Ageing induced the expression of *MAOA* (Figure 7A) but did not affect the expression of *MAOB* (Figure 7B). Although this induction suggests more oxidative stress in hearts from aged myocytes via MAO-A-dependent degradation of 5-HT, it is unlikely that this really occurs. First, the expression of the 5-HT uptake transporter (*SLC6A4*) decreased (Figure 7C), as well as that of *CAT* required for detoxification of hydrogen peroxide (Figure 7F). Second, the expression of semicarbazide-sensitive amine oxidase (*SSAO* = AOC3), an enzyme that may alternatively degrade 5-HT, declines (Figure 7G). Third, the expression of HTR2A declines during ageing (Figure 7D), whereas the expressions of HTRB2 and *ALDH2* remained unchanged (Figure 7E,H).

**Figure 8:**
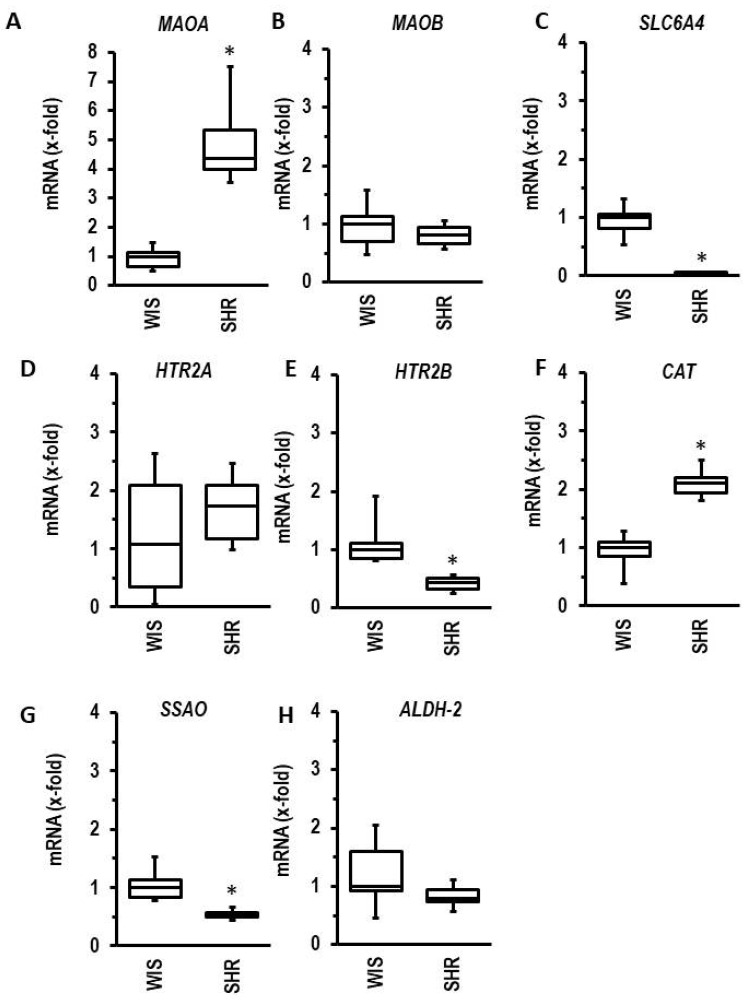
Hypertension-dependent regulation of left ventricular mRNA expression of genes involved in 5-HT metabolism or signaling ((**A**): *MAOA*, (**B**): *MAOB*; (**C**): *SLC6A4*; (**D**): *HTR2A*; (**E**): *HTR2B*; (**F**): *CAT*; (**G**): *SSAO*; (**H**): *ALDH2*). Left ventricular tissue was dissected from normotensive rats (WIS) or spontaneously hypertensive rats (SHR) at the age of 7.5 months (*n* = 6 each). *T*-tests or Mann–Whitney U-tests where appropriate. * *p* < 0.05 vs. WIS.

Similarly, the induction of hypertension (SHR vs. Wistar rats) displayed the same pattern of regulation, except for the regulation of 5-HT receptors (Figure 8).

### 3.7. Effect of MAO-A on Norepinephrine-Dependent Cell Damages

**Figure 9:**
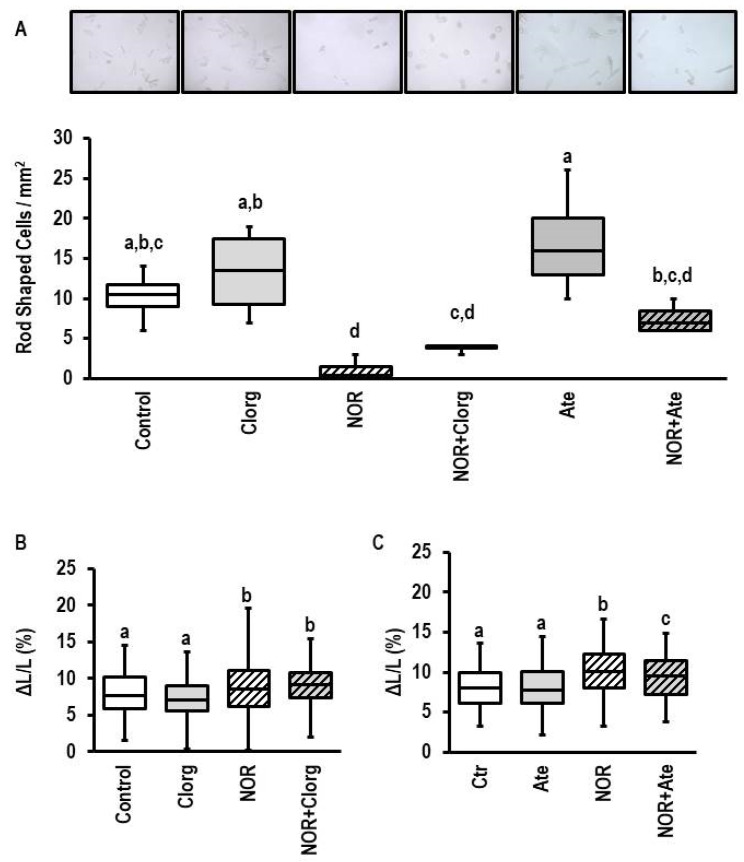
Effect of norepinephrine on cell structure and load-free cell shortening. (**A**) Original photographs obtained from cultures exposed to clorgyline (1 µM), norepinephrine (NOR, 10 µM), atwenolol (10 µM), or combinations thereof for 24 h (*n* = 4 each). For better visibility of rod-shaped and round cells see Supplementary Information. Data are expressed as the number of rod-shaped cells per mm^2^. Data are full ranges (whiskers) with median and 25 and 75% quartiles (boxes). One-way ANOVA with Student–Newman–Keuls post hoc analysis. Equal letters indicate no group differences. (**B**) Load-free cell shortening (see Figure 1 for details) for cells exposed to clorgyline (1 µM), norepinephrine (NOR, 10 µM), or combinations thereof for 24 h (*n* = 119–123). One-way ANOVA with Student–Newman–Keuls post hoc analysis. Equal letters indicate no group differences. (**C**) Load-free cell shortening (see Figure 1 for details) for cells exposed to atenolol (Ate, 10 µM), norepinephrine (NOR, 10 µM), or combinations thereof for 24 h (*n* = 97–121). One-way ANOVA with Student–Newman–Keuls post hoc analysis. Equal letters indicate no group differences.

Similar to 5-HT, excessive norepinephrine decreased the number of rod-shaped cells (Figure 9A; effect size 3.640 (CI: 1.160–6.037; *p* = 0.002). However, in contrast to 5-HT, inhibition of MAO-A by clorgyline did not protect cells against norepinephrine-dependent damaging (Figure 9A; effect size 2.751 (CI: 0.648–4.760; *p* = 0.008). In contrast, atenolol protected against norepinephrine-dependent cell damage (Figure 9A; effect size: 1.018 (−0.513–2.479; *p* = 0.200). The remaining nondamaged myocytes showed an improved load-free cell shortening in the presence of norepinephrine (Figure 9B; effect size 0.760 (CI: 0.493–1.025); *p* < 0.0001). This effect was not affected by clorgyline either (Figure 9B). However, inhibition of β-adrenoceptors by atenolol reduced the norepinephrine effect on load-free cell shortening (Figure 9C; effect size: 0.547 (CI: 0.279–0.814; *p* = 0.000061).

## 4. Discussion

This study was performed to improve our understanding about the contribution of the 5-HT metabolizing enzyme MAO-A to cardiac hypertrophy and function. Induction of *MAOA* has been shown before in several studies as a response to hypoxia, volume load, ageing, or pressure load [37,38,39,40,41,42]. Similarly, 5-HT has been associated with myocardial hypertrophy and heart failure. However, as outlined in the introduction, the contribution of 5-HT to heart failure is challenging to understand. Several authors suggest that low concentrations of 5-HT improve cardiac function via 5-HT receptor activation, whereas high concentrations of 5-HT contribute to heart failure via activation of MAO-A [11,13]. Similarly, MAO-B contributes to heart failure [8,26]. However, both isoforms show species-dependent differences in the cardiac expression [16,43]. There are still open questions: Is *MAOA* upregulated before the transition of cardiac hypertrophy to heart failure? Is *MAOA* upregulated similarly in both ventricles? Can upregulation of *MAOB* in rats occur due to pressure overload as part of a fetal reprogramming? These questions were addressed in this study.

Initially we investigated, in left and right ventricular models (AOB, PAB), whether *MAOA* and *MAOB* are differentially regulated. The new finding of our study is that in both cases, *MAOA* is specifically upregulated at the time of decompensation, whereas *MAOB* expression is not affected (results are reported in Figure 1). These data suggest that *MAOA* but not *MAOB* plays an important role in heart failure. This hypothesis was tested next. We first confirmed the lack of *MAOB* expression in rat myocardium. Subsequently, we showed, by using an MAO-B-specific substrate (PEA), that MAO-B activation neither induces cell dysfunctions in cardiomyocytes from mice nor from rats (results reported in Figure 2). In contrast, experiments using an MAO-A-specific substrate (5-HT) showed a small effect on cardiomyocytes from mice at very high concentrations that was absent in *MAOB* knockout cells. This suggests that the small effect requiring high 5-HT concentrations in mice is mediated by *MAOB* to which 5-HT has a low affinity. The data are consistent with former experiments in which deletion of *MAOA* in mice increased the plasma concentration of 5-HT, leading to excessive myocardial hypertrophy [44]. However, the *MAOA*-specific substrate 5-HT strongly reduced cell shortening in rat myocytes with high expression of *MAOA*. This conclusion is remarkable as the effect was already seen at 5-HT concentrations that are in the range of plasma 5-HT concentrations of rodents (i.e., 24 µM reported by [44]). Furthermore, the effect was attenuated by copresence of an MAO-A inhibitor (clorgyline; results reported in Figure 3). The new aspect that these experiments add to the current standing of the literature is that we show chronic effects of 5-HT on myocytes that allow a mechanistic link between the aforementioned speculation that MAO-A contributes to heart failure because it is induced in the decompensated phase, and the functional effect.

Detrimental effects of MAO-A activity are often explained by oxidative stress [12,21,22,45]. We therefore addressed the question of whether MAO-A activity is associated with oxidative stress in rat myocardium, too. First, we measured hydrogen peroxide production of isolated mitochondria exposed to 5-HT and confirmed such an effect as previously shown [11]. Importantly, the concentration–response curve (Figure 4) fits the detrimental effects shown on cardiomyocytes in our study. Furthermore, when cells were exposed to tempol, an SOD mimetic that produces hydrogen peroxide, a similar time-dependent effect on cell shortening was obtained as well. The data support the view that MAO-A activity contributes to heart failure via oxidative stress. Interestingly, although PEA induces ROS formation in isolated mitochondria from mouse hearts [26], PEA did not affect load-free cell shortening in mice myocytes, as found by selective stimulation of MAO-A.

Our own data (Figure 1), as well as previous studies performed by others, shows a similar upregulation of *MAOA* in left and right ventricles due to pressure overload in the decompensated phase [40,41]. However, we have shown before that both ventricles differ in their oxidative stress defense strategy [46]. Therefore, it is important not only to show that *MAOA* is induced in both ventricles but also to show that cardiomyocytes from both ventricles behave similarly when exposed to 5-HT. These new findings (Figure 6) indicate that there are no differences between both ventricles with respect to 5-HT responsiveness.

Induction of MAO-A during ageing has been shown before [47]. Here, we confirmed such findings on the gene level. In extension to previous studies, we compared the regulation of *MAOA* with that of other genes encoding proteins involved in 5-HT biology. The most interesting observation is that the expression of the 5-HT transporter *SLC6A4* is reduced during ageing (data shown in Figure 7). Similarly, we found the same expression profile in spontaneously hypertensive rats in comparison to normotensive rats (data shown in Figure 8). This suggests that alterative substrates other than 5-HT are metabolized by MAO-A under such conditions. An attractive alternative are catecholamines. Therefore, we finally investigated whether norepinephrine exerts MAO-A-dependent effects in adult cardiomyocytes. Recently, MAO-A upregulation was identified as part of β-adrenoceptor desensitization by reducing the intracellular concentration of norepinephrine needed for interaction between β-adrenoceptors and the phospholamban/SERCA2a complex at the SR [24]. Norepinephrine damaged ARVM, as indicated by loss of rod-shaped structure. However, unlike the effect on 5-HT, this effect was not blunted by pharmacological MAO-A inhibition (data shown in Figure 9). The remaining nondamaged myocytes displayed an improved load-free cell shortening in contrast to nondamaged 5-HT treated myocytes, which displayed a reduced cell shortening. Again, this effect of norepinephrine was not attenuated by clorgyline, the MAO-A inhibitor. However, administration of a β-blocker (atenolol) normalized partly cell shortening of nondamaged myocytes. These data clearly show that norepinephrine acts via receptor-dependent pathways, whereas 5-HT acts via MAO-A.

## 5. Conclusions

Our study couples the expression of *MAOA* in the decompensated phase of myocardial hypertrophy to direct damaging effects of MAO-A activity. Using 5-HT as a more or less specific MAO-A substrate, we showed that high activity of MAO-A reduces cell function (load-free cell shortening) and leads to structural damages in cells (loss of rod-shaped morphology). We also showed that norepinephrine induces structural damage but in a receptor-dependent and not MAO-A-dependent way. Norepinephrine does not affect cell shortening under these conditions. Finally, we showed that MAO-A activity induces hydrogen peroxide and that a hydrogen-peroxide-forming molecule induces a comparable effect. This suggests that the MAO-A-dependent effects seen here with 5-HT are performed via oxidative stress. The open questions that require future studies are: Why do genetically related species such as mice and rats express different MAO isoforms? What are the main substrates of MAO-A in stressed hearts, if neither 5-HT metabolism (as suggested by decreased expression of *SLC6A4* and increased expression of *CAT*) nor norepinephrine (an alternative substrate of MAO-A) are proper candidates? How is MAO-A linked to glucose metabolism, and is there a linkage with other molecules of the inner mitochondrial membrane, such as UCP2? Future studies are required to shed light on these open questions.

## Data Availability

Original data can be requested from the corresponding author.

## Supplementary Materials

The following supporting information can be downloaded at: https://www.mdpi.com/article/10.3390/biom13061013/s1, Figure S1: Study Plan; Table S1: Primer List.
