# Supplementary material for: Monoamine Oxidase A Contributes to Serotonin—But Not Norepinephrine-Dependent Damage of Rat Ventricular Myocytes
Source: Biomolecules. 2023 Jun 19;13(6):1013. doi: 10.3390/biom13061013 (PMC10296391; doi:10.3390/biom13061013)
Supplement: Supplementary file 1 [file biomolecules-13-01013-s001.zip › Supplement Figure S1.pptx]

## Slide 1
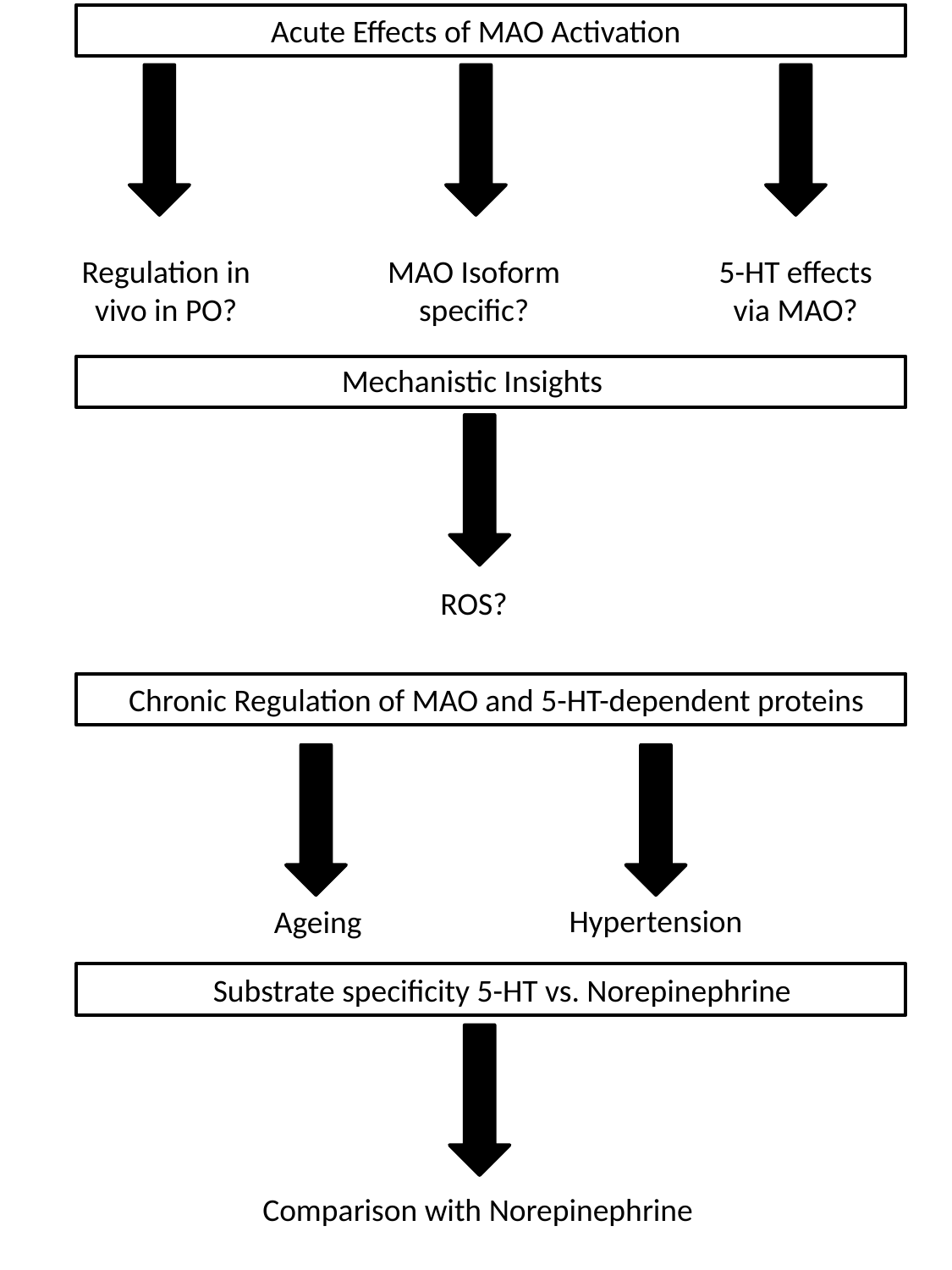

Acute Effects of MAO Activation
MAO Isoform specific?
Regulation in vivo in PO?
5-HT effects via MAO?
Mechanistic Insights
ROS?
Chronic Regulation of MAO and 5-HT-dependent proteins
Hypertension
Ageing
Substrate specificity 5-HT vs. Norepinephrine
Comparison with Norepinephrine
