# Supplementary material for: Monoamine Oxidase A Contributes to Serotonin—But Not Norepinephrine-Dependent Damage of Rat Ventricular Myocytes
Source: Biomolecules. 2023 Jun 19;13(6):1013. doi: 10.3390/biom13061013 (PMC10296391; doi:10.3390/biom13061013)
Supplement: Supplementary file 1 [file biomolecules-13-01013-s001.zip › Supplement Table S1.pdf]

Supplement Table S1: List of primer sequences used in this study

Gene	Forward	Reverse	Reference
ALDH2	TGGCTGATCTCATCGAACGG	CAGCCAGCATAATAGCCGGAGA	NM_032416.1
AOC3	GCGAGAAGTTTGGGAACCGA	TTCTTCAACCCTGCCACATCC	NM_031582.3
B2M	GCCGTCGTGCTTGCCATTC	CTGAGGTGGGTGGAACTGAGAC	NM_012512.2
CAT	GCCCCTCCTCGTTCAAAGATG	TATCCAAAAGCACCTGCTCCC	NM_012520.2
HPRT	CCAGCGTCGTGATTAGTGAT	CAAGTCTTTCAGTCCTGTCC	NM_012583.2
HTR2A	TGGTCATCATGGCAGTGTCC	CCACCGGTACCCATACAGGA	NM_017254.1
HTR2B	ATGTTTGAGGCTACATGGCCC	CGGGAATTGCACTGATTGGC	NM_017250.2
MAOA	GTTGGACAAAAACTGCTCGGG	GGAACCACAGGGCAGATACC	NM_033653.1
MAOB	GGCTGTGCTGCTATAATGGGA	ATAATGGACTGGCTGCAGAGC	NM_013198.1
RPL32	CAGGGTGCGGAGAAGATTCA	TCTCAGCACAGTAAGATTTGTTTGC	NM_013226.3
SLC6A4	AGGAGTTCTACTTGCGCCAT	TTGCCAGATGTTTTGACGCC	NM_013034.4
